# Gleanings from the French

**Published:** 1875-01-15

**Authors:** 


### GLEANINGS FROM THE FRENCH.

*Translated by Fred. J. Huse, M.D.*

IN a note to the editor of *La France Medicale*, Prof. Bouchut calls attention to the use of the ophthalmoscope as a valuable means of diagnosis in traumatic injuries of the brain.

In simple concussion of the brain, the ophthalmoscope fails to indicate the existence of any lesion of either the optic papilla or the retina and retinal vessels. The condition of the fundus is nearly normal.

In contusion, however, followed by encephalitis, and in compression of the brain, the appearance of the fundus affords a measure of the effect produced within the cranium. The papilla of the optic nerve is red, swollen, of a flattened appearance, and more or less obscure and diffuse on account of the hyperæmia of the nerve. It is sometimes the seat of an œdema which extends throughout the neighboring portion of the retina, and gives rise to an acute neuro-retinitis.

In addition, the retinal veins enlarged, flexuous, and sometimes filled with thromboses, thus indicating the difficulty with which the blood re-enters the cranium from the fundus; or an effusion obstructing the progress of the blood in the sinuses of the dura mater.

---

ACCORDING to Dr. Thorowgood, the true active principle of arnica is trimethylamine, which though only slightly soluble in alcohol, is readily extracted by water. An additional fact in favor of an aqueous solution, is its failure to extract an irritant oil, which is usually present in the alcoholic solution.

